# Color Trails Test: regression-based norms for Russian-speakers

**DOI:** 10.1093/arclin/acag035

**Published:** 2026-05-20

**Authors:** Zarui A Melikyan, Jason R Bock, Anna V Agranovich

**Affiliations:** Department of Neurobiology and Behavior, University of California, Irvine, CA, USA; Department of Neurobiology and Behavior, University of California, Irvine, CA, USA; Department of Physical Medicine and Rehabilitation, Johns Hopkins University School of Medicine, Baltimore, MD, USA

**Keywords:** Color Trails Test, CTT norms, culture-sensitive norms, regression-based norms, norms for Russian-speakers

## Abstract

**Objective:**

The Color Trails Test (CTT) is widely used across cultures and necessitates culture-relevant norms due to cultural differences in the test performance. However, no norms exist for Russian-speakers. The study aims to: (a) establish regression-based CTT norms for Russian-speaking adults, and (b) compare CTT T-scores for Russian-speakers derived using regression-based norms (RBNs) with the manual norms.

**Method:**

Community-dwelling healthy Russian-speaking adults were administered the CTT using standard procedures. Multiple linear regression analysis was applied to establish RBNs. The participant data were used to calculate two sets of T-scores, using: (a) RBNs and the leave-one-out analysis, and (b) CTT manual norms. These two sets of T-scores were compared by *t*-test.

**Results:**

The mean age of the 196 participants was 37 years [range: 18–86]; 108 (55%) were women, and 102 (52%) had at least university education. CTT completion time was associated with age and education, but not sex. CTT1 T-scores derived from regression-based were higher than manual norms (*M* difference = 2.39, *SD* difference = 5.51, *p* < .001). There was no difference between CTT2 T-scores. Regression-based norms identified fewer participants as performing below average (T ≤36): 7% vs. 18% for CTT1 and 7% vs. 10% for CTT2, *p* < .001.

**Conclusions:**

The proposed RBNs, developed from a representative sample of Russian-speaking adults, will enhance assessment accuracy and improve diagnostic outcomes.

## Introduction

The Color Trails Test (D’Elia et al., 1996) is a frequently used neuropsychological assessment tool aimed to assess visual attention, scanning, mental flexibility, and motor speed. It is considered a culture-fair analog of the Trail Making Test (TMT) (D'Elia et al., 1996; Elkin-Frankston et al., 2007; Hartman-Maeir et al., 2008; Maj et al., 1993; Rabin et al., 2005) because it relies on the presumably universal Arabic number sequence and discrimination of colors instead of the language-based alphabet used in the original TMT. This assumption prompted application of CTT in a wider cultural and linguistic context, including non-English speakers (Avila et al., 2019; Elkin-Frankston et al., 2007; Puente et al., 2013). However, elimination of the alphabet did not remove other cultural factors, such as familiarity with testing procedures, attitudes toward timed tests, and importance placed on speed versus accuracy, which have been shown to contribute to cultural differences in the neuropsychological test performance (Ardila, 1995; Ardila et al., 2002).

Indeed, studies have reported longer CTT completion time in various cultural groups when compared to U.S. samples (Agranovich et al., 2011; Agranovich & Puente, 2007; Fasfous et al., 2013; Melikyan et al., 2021). This necessitated development of culture-specific CTT norms. To-date, CTT norms have been published for Chinese (Hsieh & Tori, 2007), Greek (Konstantopoulos et al., 2013; Messinis et al., 2011; Staios et al., 2023), Hispanic and Latino (La Rue et al., 1999; Pontón et al., 1996), Turkish (Kudiaki & Aslan, 2008), French-Speaking Canadians (Gaudreau et al., 2022), Danish (Vogel et al., 2013) Brazilian (Sant’ Ana Rabelo et al., 2010), Arabic (Al-Joudi et al., 2019), Iranian (Avila et al., 2019; Tavakoli et al., 2015), and Indian (Indorewalla et al., 2022) samples. However, no CTT norms have yet been developed for Russian-speakers, despite the presence of large Russian-speaking populations across multiple countries around the world. According to the American Community Survey, Russian is among the top 15 most spoken languages in the United States, with almost a million people speaking Russian at home (Dietrich & Hernandez, 2019).

The need to develop CTT norms for Russian-speaking individuals arises from research showing that Russian-speakers perform more slowly on CTT1 and CTT2 compared to demographically matched U.S. peers (Agranovich et al., 2011; Agranovich & Puente, 2007; Melikyan et al., 2021). Hence, using U.S. norms to evaluate Russian-speaking individuals may lead to misclassification and diagnostic errors, similarly to findings reported with other cultural groups (Avila et al., 2019; Fasfous et al., 2013; Ferrett et al., 2014). These performance discrepancies have been attributed to cultural differences in attitudes toward time and varying levels of experience with standardized, timed tests (Agranovich et al., 2021). In the Soviet Union, and in the post-Soviet space for decades after its collapse in 1991, there was little emphasis on deadlines, speed of performance, or timeliness in the educational system or broader cultural environment. Additionally, familiarity with standardized, timed achievement tests was limited, as such assessments only began to be introduced in the region in the early 2000s.

There are two main approaches to the development of test norms: the conventional approach, where an individual’s test score is compared to that of demographically-matched peers, and the regression-based approach, which uses multiple regression analysis to predict an individual’s test score based on one’s unique demographic characteristics. The regression-based approach has several advantages. First, regression-based norms (RBNs) enable prediction of test performance based on the unique combination of demographic characteristics for each individual, making normative conversions highly individualized. In contrast, in the conventional norms approach, close matching of an individual’s demographic characteristics with that of a normative group is not always possible, as norms are often based on limited characteristics (e.g., only by age, or only by education) or have insufficient demographic granularity (e.g., norms are presented just for two age categories: children vs. adults). Second, the regression-based approach does not require a large sample size, and 15–20 individuals per independent variable are considered sufficient to derive reliable equations (Stevens, 2002), though statistical assumptions must be met to ensure stability and reliability of regression equations. In contrast, the conventional approach of stratifying normative group into demographic subgroups requires a larger sample size for adequate reliability. Third, RBNs can account for non-linear and interaction effects of demographic variables on test performance. The conventional approach does not easily allow for that because participants are assigned, at times arbitrarily, to certain demographic categories.

The present study aims to (a) establish regression-based CTT norms that incorporate relevant effects of demographic variables on test performance for Russian-speakers; and (b) demonstrate the utility of these RBNs for the evaluation of Russian-speakers, by comparing T-scores derived from RBNs with those obtained from the CTT manual norms.

## Materials and methods

### Study procedures

The normative sample was derived from three separate cross-cultural studies, where performance on North American neuropsychological tests were assessed cross-sectionally in healthy community-dwelling Russian-speaking adults living in Russia (Agranovich et al., 2011; Agranovich & Puente, 2007; Melikyan et al., 2021). Data collection for the three studies was led by the first and senior authors between 2003 and 2014. The studies shared similar recruitment strategies, procedures, and exclusion criteria. Participants were recruited through word of mouth and local advertisements in several urban and rural areas of European Russia. All testing procedures were completed in Russian. Tests were administered either by psychometrists with a Bachelor’s or Master’s degree in Psychology, who were trained in standardized testing procedures according to the test manual. Test administrations were recorded, reviewed, and closely monitored via regular communication. All participants completed CTT1 and CTT2 trials. The raw scores included the completion time (in seconds) and the total number of errors for each of the two subtests (CTT1 error data were missing for 40 participants, and CTT2 error data were missing for 41 participants).

### Participants

For the three studies, native Russian-speaking community-dwelling adults aged 18–89 years were screened using a general health questionnaire (Agranovich et al., 2011) prior to engaging in the study. Individuals with a reported history of neurological, psychiatric or developmental disorders, substance abuse, or color blindness were excluded. The combined sample from the three studies includes a broad range of educational and occupational categories, with both men and women equally represented.

The studies that contributed to this normative dataset were approved by the respective Institutional Review Boards. All participants signed written informed consent in Russian. The research was completed in accordance with the Helsinki Declaration.

### Assessments

#### Predictors

The predictors were demographic variables, including age, sex, and education level. Studies have shown that using years of education may be problematic in cross-cultural research as education systems and years at each education level may vary significantly across cultures and age/cohorts (Ardila, 2005; Manly et al., 2003; Manly & Echemendia, 2007). Specifically, considering the changes over the past decades in the Russian educational system (e.g., secondary education length changed several times between 10 and 11 years; a university degree length changed over the decades from 5–6 years to 4 + 2 years in some institutions, while maintaining comparable curricula), reporting years of education may lead to the misclassification of an individual’s level of education or degree. Hence, the education variable was categorized as: (a) below university degree, and (b) university degree and beyond (a university degree in Russia is typically equivalent to a Master’s or professional [e.g., MD, JD] degree in the United States).

#### Outcome

The outcome was the CTT completion time in seconds for two parts: CTT1 and CTT2. On CTT1, the task is to connect the circles numbered from 1 to 25 in numerical order, regardless of the color of the circle. On CTT2, each number after 1 is presented twice, once in a pink circle and once in a yellow circle. The task is to draw a line connecting the circles in numerical order while alternating between the yellow and pink circles. The respondent must be able to recognize Arabic numerals and distinguish between pink and yellow colors. The test developers suggested that even a colorblind individual would be able to detect the difference between colors on the basis of darkness (D'Elia et al., 1996). However, in an abundance of caution, color-blind individuals were not included in this sample.

Several types of errors are recorded for the CTT1 (near-misses, prompts) and CTT2 (color errors, number errors, near-misses, prompts) tests. This study provides the total number of errors for CTT1 and CTT2.

#### CTT translation and cultural considerations

The CTT was chosen for the assessment of Russian-speakers because of its reported applicability across cultures. Thus, it is suggested that the CTT (a) has the same meaning of test scores and measures the same psychological constructs with equal accuracy in different cultural groups (Maj et al., 1993); (b) provides comparable assessment of cognitive abilities across environments (Ardila et al., 2002; Helms, 1997; Nell, 2012; Puente et al., 2013; Puente et al., 2015); and (c) is widely used by neuropsychologists (Rabin et al., 2016).

Culturally-sensitive translation and adaptation of the CTT into Russian was based on published guidelines (American Educational Research Association, et al., 2014; Ardila, 1995; Ardila et al., 1989; International Test Commission, 2017), which recommend: (a) using experts with sufficient knowledge of the languages involved, the cultures, the test content, and general testing principles; (b) maximizing the suitability of adapted tests to the target population and focusing on functional rather than literal equivalence by using forward and backward translation of test materials, and subsequent reconciliation to address discrepancies of the original and back translated versions; (c) ensuring that test instructions and item content have similar meaning and familiarity across cultures. For the original studies, test instructions were translated from English into Russian by the authors of this manuscript, who are Russian–English bilingual neuropsychologists educated both in Russia and in the United States. Tests were back-translated from Russian into English by Russian–English bilingual psychologists, who were educated in Russia and the United States. or Canada, and by a linguist educated in Russia and the United States. The English translation was then compared with the original English instructions. Discrepancies were addressed individually according to conceptual, functional, and metric equivalence (American Educational Research Association et al., 2014).

### Statistical analyses

#### Establishing regression-based Russian norms

To establish RBNs for CTT performance with the relevant predictors of age, sex, and education, we followed the methodology outlined by Testa and colleagues (2009). Briefly, the RBN approach uses multiple regression analysis to model cognitive test scores (outcome) as a function of demographic variables (predictors). The resulting equations can be used to predict test performance for the specific individual’s demographics, rather than for groups that are merely demographically similar to the individual. In the model, each predictor has an estimated coefficient that indicates the change in the outcome corresponding to a one-unit change (e.g., 1 year of age) or a categorical difference from the reference (e.g., university degree or higher education group relative to less than university degree group as a reference). The accuracy of the model depends on the proportion of variance in the outcome that is explained by the predictors. Residual variance accounts for the difference between predicted and actual outcome values in the normative sample. For a new participant who is not in the normative sample, the residuals can be used to estimate the likelihood of their observed score by taking the difference between their normative model-predicted score and their observed score and dividing that difference by the standard error of the estimate (*SEE*). This produces a *z*-score that can be linearly scaled to a T-score.

Prior to evaluating the regression models, the outcomes—raw CTT1 and CTT2 completion time in seconds—were transformed to normalized scaled scores by obtaining the quantile function of the cumulative frequency distribution of the ranks within the sample and linearly scaling the resulting scores (*M* = 10, *SD* = 3, range: 1–19). Scores were reversed to ensure that higher completion time corresponded to a lower scaled score and percentile range. For each of the CTT1 and CTT2 scaled scores as outcomes, we evaluated a series of regression analyses to identify the best-predicting models. Initial models included demographic predictors (age, education, and sex) and their interactions (squared age, age × education, age × sex, and age × education × sex). A backward elimination approach was applied for the stepwise removal of the least-contributing predictors with likelihood-ratio test evaluation. Statistical assumptions for regression analyses were assessed with Levene’s test for homogeneity of variance, pairwise correlations for multicollinearity among predictor variables, and q-q plots for homoskedasticity of residuals, in all cases meeting satisfaction.

To evaluate the influence of settlement type (i.e., rural vs. urban) on CTT performance, we conducted supplementary analysis using data from a subset of participants (*n* = 101, 52% of the total sample: *n* = 52, 27% rural; *n* = 49, 25% urban), for which information on settlement type was available. Age, sex, and education were compared between settlement types with an independent samples *t*-test and chi-square tests, respectively. We added settlement as a predictor to the multiple regression models presented on the full sample.

#### Comparison of regression-based Russian norms to CTT manual norms

The final regression models obtained from the full sample were subsequently applied in prediction of individual participants within the sample using a leave-one-out analytical approach (Hastie et al., 2009), separately for CTT1 and CTT2. All but one participant were evaluated with a multiple regression analysis to estimate predictor coefficients and *SEEs* as described earlier, and the remaining one participant’s observed score was compared to their predictions with that model. As would be done for identifying a participant outside of the normative sample, the T-score for that one participant was obtained. This was then repeated sequentially for all participants, obtaining T-scores for the full sample. The goodness of fit for the regression model was evaluated with a one-sample *t*-test against a mean of 50. A result significantly different from 50 was considered to systematically under- or over-predict and prompted reevaluation of the model.

To assess the utility of these Russian RBNs over the CTT manual norms for the evaluation of Russian-speakers, T-scores derived from the leave-one-out analysis were comparted to T-scores obtained with the demographically-stratified tables provided in the CTT manual (D'Elia et al., 1996). For both CTT1 and CTT2, differences between T scores obtained through the Russian RBN and the CTT manual within participants were assessed using paired-samples *t*-tests. A significant difference was considered to indicate a better fit with the Russian RBN than the CTT manual scores for Russian-speakers. To assess differences in meaningful classification ability, a T-score threshold of 36 was used, corresponding to the below average range according to the American Academy of Clinical Neuropsychology (AACN) guidelines (Guilmette et al., 2020). Frequencies of below average performance identified with both manual-based and Russian-sample-derived T-scores were evaluated using chi-square tests for independence. A significant difference was considered to indicate the misclassification of Russian participants when using the manual-based T-scores.

Normative values for the CTT1 and CTT2 number of errors are presented as cumulative percentiles only and did not undergo comparison with the scores derived using the U.S. manual.

Participant characteristics are reported as numbers and percentages for categorical variables, and as means, standard deviations (*SDs*), and ranges for continuous variables.

All analyses were considered significant at an alpha of 0.05 and were performed using SAS 9.4 (SAS Institute Inc., Cary, NC) and Python (v3.13.1) libraries SciPy (v1.15.2) and statsmodels (v0.14.4).

## Results

### Participant characteristics

The normative sample consisted of 196 Russian-speaking adults with a mean age of 37 years (*SD* = 17, range: 18–86). Of those 55% (108) were women, and 52% (102) had university education or higher (Table 1; see Supplementary material, online Fig. S1 for age distribution by decade).

**Table 1:** Participant characteristics.

	***N* (%)**
*N*	196 (100)
Sex	
Men	88 (45)
Women	108 (55)
Education	
Below university	94 (48)
University or higher	102 (52)
	**Mean (*SD*) [range]**
Age, years	37.2 (16.7) [18.0–86.0]

*Note*: University education in Russia is equivalent to a master’s or professional (e.g., MD, JD) degree.

### Regression-based norms for CTT completion time

**Figure 1:**
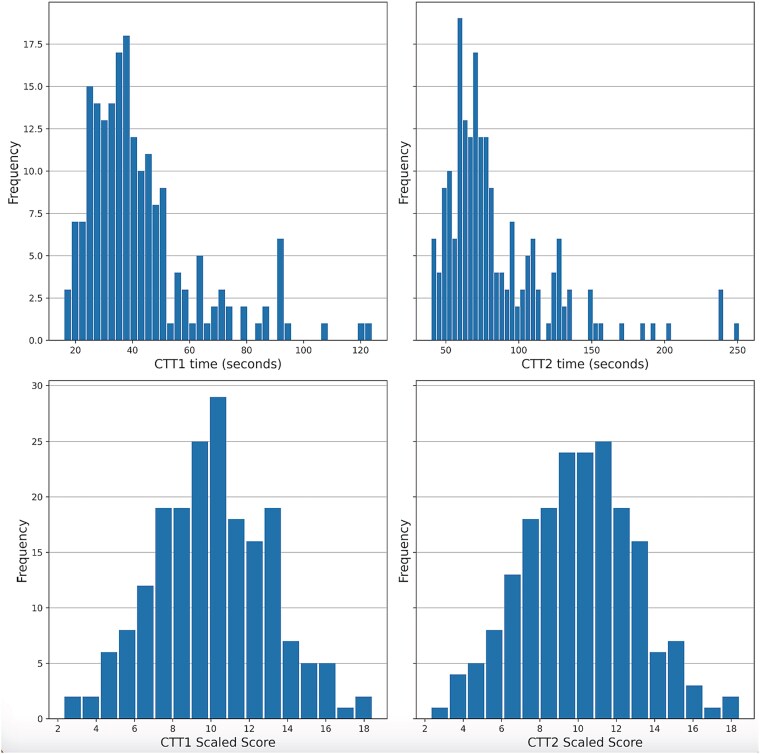
Distribution of raw CTT1 and CTT2 (upper panels) and scaled scores (lower panels). CTT = Color Trials Test.

Completion time raw scores (CTT1 *M* = 42.77, *SD* = 19.62; CTT2 *M* = 84.25, *SD* = 37.69) were transformed to scaled scores (CTT1 *M* = 10.05, *SD* = 2.99; CTT2 *M* = 10.05, *SD* = 2.99) for use as outcomes in the regression analyses (one participant had a missing CTT2 score and was excluded from the CTT2 analysis only). Extreme values were retained for analyses, as these belonged to older participants and were considered representative of the normative population. See Table 2 for conversion between raw scores, cumulative percentiles, and scaled scores, and Fig. 1 for distributions of raw and scaled scores.

**Table 2:** Conversion among raw scores, cumulative percentiles, and scaled scores.

**Scaled score**	**CTT1**		**CTT2**		**Scaled score**
	**Raw score**	**Cumulative percentile**	**Raw score**	**Cumulative percentile**	
19	—	—	—	—	19
18	<16.0	≥98.99	<40.0	≥98.98	18
17	16.0–17.9	98.98–99.73	40.0–40.9	98.97–99.73	17
16	18.0–19.9	97.19–98.97	41.0–42.9	97.95–98.96	16
15	20.0–21.9	94.39–97.18	43.0–48.9	93.33–97.94	15
14	22.0–24.9	88.27–94.38	49.0–50.9	88.97–93.32	14
13	25.0–26.9	82.14–88.26	51.0–57.9	81.28–88.96	13
12	27.0–30.9	71.17–82.13	58.0–62.9	70.51–81.27	12
11	31.0–34.9	60.20–71.16	63.0–68.9	58.46–70.50	11
10	35.0–39.9	44.64–60.19	69.0–75.9	44.36–58.45	10
9	40.0–44.9	31.89–44.63	76.0–86.9	31.54–44.35	9
8	45.0–50.9	20.41–31.88	87.0–104.9	21.28–31.53	8
7	51.0–63.9	13.27–20.40	105.0–124.9	13.08–21.27	7
6	64.0–79.9	7.14–13.26	125.0–135.9	7.18–13.07	6
5	80.0–90.9	4.85–7.13	136.0–184.9	3.59–7.17	5
4	91.0–107.9	1.53–4.84	185.0–238.9	2.05–3.58	4
3	108.0–119.9	1.02–1.52	239.0–239.9	1.28–2.04	3
2	≥120.0	<1.02	≥240.0	<1.28	2
1	—	—	—	—	1

*Note*: CTT = Color Trials Test.

**Figure 2:**
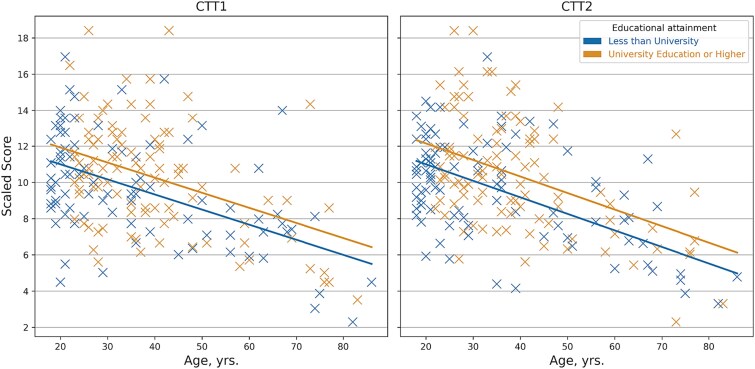
Model-predictive slopes and observed scaled scores.

The full model, with all demographic predictors and their interactions, significantly predicted CTT1 (*R*^2^ = 0.251) and CTT2 (*R*^2^ = 0.297) scaled scores, and backward elimination sequentially excluded predictors until a final model was obtained with only predictors of education (below university degree vs. university degree or higher) and squared age years retained (CTT1 *R*^2^ = 0.244, *SEE* = 2.619; CTT2 *R*^2^ = 0.285, *SEE* = 2.549 all *p*’s < .001). However, upon performing the leave-one-out analysis to evaluate goodness of fit, the models that predicted a curvilinear decline from younger to older age were found to systematically over-predict participants performance, with T-scores of CTT1 (*M* = 44.13, *SD* = 11.72) and CTT2 (*M* = 43.45, *SD* = 11.82) for the testing set significantly below 50, using one-sample *t*-tests (*t*’s = −7.00 and −7.72, respectively; *p*’s < .001). Therefore, we excluded squared age years as the final predictor removal step, and retained linear age years instead, resulting in final models that significantly predicted CTT1 (*R*^2^ = 0.225, *SEE* = 2.651; *p* < .001) with education (*b* = 0.928 [95% CI = 0.176, 1.680], *p* = .016) and age (*b* = −0.083 [95% CI = −0.106, −0.061], *p* < .001); and CTT2 (*R*^2^ = 0.268, *SEE* = 2.578; *p* < .001) with education (*b* = 1.150 [95% CI = 0.416, 1.884], *p* = .002) and age (*b* = −0.091 [95% CI = −0.113, −0.069], *p* < .001). Leave-one-out analysis confirmed goodness of fit with no significant differences between predicted participants’ T-scores and 50 for either CTT1 (*M* = 49.87, *SD* = 10.39, *t* = −0.18, *p* = .857) or CTT2 (*M* = 49.78, *SD* = 10.16, *t* = −0.30, *p* = .763). See Table 3 for regression coefficients and Fig. 2 for model-predictive slopes and observed scaled scores.

**Table 3:** Multiple regressions predicting CTT1 and CTT2 scaled scores.

**Coefficient**	**CTT1, *N* = 196**		**CTT2, *N* = 195**	
	**SS (95% CI)**	***p*-value**	**SS (95% CI)**	***p*-value**
Intercept	12.666 (11.703, 13.628)	<.001	12.827 (11.888, 13.765)	<.001
Education, less than university degree	Ref		Ref	
Education, university degree or greater	0.928 (0.176, 1.680)	.016	1.150 (0.416, 1.884)	.002
Age, years	−0.083 (−0.106, −0.061)	<.001	−0.091 (−0.113, −0.069)	<.001
*SEE*	2.651	—	2.578	<.001
*R* ^2^	0.225	—	0.268	<.001

*Note*: One participant was excluded from the CTT2 analysis only due to missing data; CI = confidence interval; CTT = Color Trails Test; *SEE* = standard error of the estimate; SS = Sjögren’s syndrome.

These coefficients can be used to calculate the T-score for a new participant, following these steps:

1. Obtain the scaled score for the observed CTT completion time from Table 2.
2. Calculate the RBN-predicted score using the following formula: predicted CTT1 = 12.666 + 0.928 (if university education or higher, else 0) + −0.083 × age (years).
3. Subtract the predicted score from the Table 2-derived score and divide the result by the *SEE* = 2.651, to obtain a *z*-score for the discrepancy between the actual completion time and expected completion time for the participant’s demographics.
4. Linearly scale the *z*-score by multiplying it by 10 and adding 50 to obtain a T-score.
5. For CTT2, perform the same steps, but with a different formula and *SEE*: Predicted CTT2 = 12.827 + 1.150 (if university education or higher, else 0) + −0.091 × age (years); *SEE* = 2.578.

For example, a 65-year-old individual with below a university degree education level and a CTT1 completion time of 43 s would have a scaled score of 9; a predicted scaled score of 7.271 = 12.666 + 0 + (−0.083 × 65); a difference of 1.729 = 9–7.271; a *z*-score of 0.652 = 1.729/2.651; and a T-score of 56.52 = 0.652 × 10 + 50.

### Supplementary analysis

Participants living in rural and urban dwellings (the subsample) differed by age and education but not by sex. Rural participants were older (*M* years = 49.23, *SD* = 19.25) than urban participants (*M* years = 36.53, *SD* = 14.53) (*t* = −3.73, *p* < .001), and less frequently had completed a university degree or higher (*n* = 22, 42%) compared to urban participants (*n* = 32, 65%) (χ^2^ = 5.36, *p* = .021). Women were evenly distributed across rural (*n* = 31, 60%) and urban (*n* = 28, 57%) dwellings (χ^2^ = 0.06, *p* = .801) (Supplementary material, online Table S1). Living in a rural versus urban region did not significantly predict the CTT1 scaled score (*b* = −0.954 [95% CI = −2.172, 0.264], *p* = .123) but significantly predicted a reduction in the CTT2 scaled score (*b* = −1.604 [95% CI = −2.585, −0.622], *p* = .002). For both CTT1 and CTT2 models, the inclusion of settlement type accounted for variance in education, such that education was no longer a significant predictor (Supplementary material, online Table S2).

### Comparison of T-scores derived using Russian RBN versus CTT manual norms

Leave-one-out-predicted T-scores were compared against manual-derived T-scores for CTT1 (*M* = 47.48, *SD* = 10.95) and CTT2 (*M* = 50.69, *SD* = 10.40). For CTT1 (*M* difference = 2.39, *SD* difference = 5.51), the normative sources were significantly different (*t* = 6.05, *p* < .001). For CTT2 (*M* difference = −0.91, *SD* difference = 6.55), the difference was not significant (*t* = −1.93, *p* = .055). When considering the below average T-scores of 36 and below, the CTT manual-derived and the Russian leave-one-out-predicted T-score classifications differed significantly, with 35 (18%) and 14 (7%), respectively, for CTT1 (χ^2^ [1] = 69.35, *p* < .001), and 19 (10%) and 14 (7%), respectively, for CTT2 (χ^2^ [1] = 65.26, *p* < .001) falling in the essentially impaired performance range (Table 4). In both cases, the CTT manual-derived T-scores identified more participants in the below average range than the Russian-based model predicted.

**Table 4:** Cross-tabulation of the frequencies of CTT T-scores (in >36 and ≤36 categories) derived using U.S. manual and using Russian sample model-prediction.

**U.S. manual-derived T-scores**	**Russian sample model-predicted T-scores**		**Total**	**χ** ^**2**^	***p*-value**
	**>36**	**≤36**			
	***N* (%)**				
CTT1^a^				69.35	<.001
>36	161 (82)	0 (0)	161 (82)		
≤36	21 (11)	14 (7)	35 (18)		
Total	182 (93)	14 (7)	196 (100)		
CTT2^b^				65.26	<.001
>36	172 (88)	4 (29	176 (90)		
≤36	9 (5)	10 (5)	19 (10)		
Total	181 (93)	14 (7)	195 (100)		

*Note*: Percent is of the entire sample *N* = 196 for CTT1, and *N* = 195 for CTT2 where one participant is missing CTT2 data; CTT = Color Trails Test.

a CTT1 = CTT1 T-scores with Russian sample model-prediction (*M* = 49.87, *SD* = 10.39) and U.S. manual derivation (*M* = 47.48, *SD* = 10.95).

b CTT2 = CTT2 T-scores with Russian sample model-prediction (*M* = 49.78, *SD* = 10.16) and U.S. manual derivation (*M* = 50.69, *SD* = 10.40).

χ^2^ tests of independence comparing the frequencies of T-scores in T >36 and T ≤36 categories using U.S. manual-derived T-scores and Russian sample model-predicted T-scores.

### Russian norms for the CTT number of errors

The distribution of the number of errors and their cumulative percentiles is presented in Table 5.

**Table 5:** Conversion of the number of CTT errors to cumulative percentiles.

**Errors**	**CTT1**		**CTT2**	
	**Cumulative percentile**	***N***	**Cumulative percentile**	***N***
0	≥51.60	152	≥61.29	121
1	<51.60	4	12.90–61.28	29
2	—	—	2.26–12.89	4
3	—	—	<2.26	1

*Note*: CTT = Color Trails Test.

## Discussion

This study’s innovative contribution lies in the development of regression-based CTT norms specifically for Russian-speaking adults. The need for such norms arises from documented cultural differences in CTT performance (Fasfous et al., 2013), with several studies highlighting distinct patterns in CTT performance between Russian and American adults (Agranovich et al., 2011; Agranovich & Puente, 2007; Echemendia et al., 2020; Melikyan et al., 2021). These findings underscore the importance of not relying on existing CTT manual norms when evaluating Russian-speaking individuals, despite the presumed culture-fairness of CTT.

Consistent with the original CTT normative study (D'Elia et al., 1996) and numerous reports on CTT adaptation across cultures (Avila et al., 2019; García-Escobar et al., 2024; Hsieh & Tori, 2007; Indorewalla et al., 2022; Konstantopoulos et al., 2013; Messinis et al., 2011; Sant’ Ana Rabelo et al., 2010; Staios et al., 2023; Tavakoli et al., 2015), present analyses confirmed that age was significantly associated with CTT1 and CTT2 completion time, with older participants taking longer to complete the tests, suggesting age-related decline in psychomotor speed and certain executive abilities.

Further, participants with higher level of education completed both CTT subtests more quickly than those with lower education. This pattern is also consistent with the original CTT normative study (D'Elia et al., 1996) and several normative studies across cultures and languages (García-Escobar et al., 2024; Hsieh & Tori, 2007; Indorewalla et al., 2022; Konstantopoulos et al., 2013; Messinis et al., 2011; Sant’ Ana Rabelo et al., 2010; Staios et al., 2023; Tavakoli et al., 2015). The positive association between higher educational attainment and faster CTT performance suggests that the test may capture cognitive skills that are developed and refined through education.

Also consistent with the majority of published CTT normative adaptations (García-Escobar et al., 2024; Konstantopoulos et al., 2013; Staios et al., 2023; Tavakoli et al., 2015), this study did not reveal significant association between sex and CTT completion time. A handful of studies, however, found that men were faster than women on CTT1 and CTT2 (Hsieh & Tori, 2007; Indorewalla et al., 2022; Sant’ Ana Rabelo et al., 2010), which was attributed to sample bias, smaller sample size, or, possibly, to better performance in men on a measure of attention and concentration, though these findings are yet to be replicated.

A supplementary analysis of the association of the CTT performance with the type of settlement, rural versus urban, revealed that living in a rural region significantly reduced the CTT2 scaled score, but not the CTT1 scaled score. It is likely that cognitive switching ability is superior in those from urban areas due to higher cognitive demands and opportunities for higher education achievement among urban dwellers.

In this study, average T-scores were lower when CTT manual norms were applied than when Russian-speaker norms were used for the Russian sample. This is consistent with previous reports that Russian-speakers took longer to complete the CTT compared to U.S. samples (Agranovich et al., 2011; Agranovich & Puente, 2007; Echemendia et al., 2020; Melikyan et al., 2021). Similar findings—that the U.S. participants perform faster on CTT—have been reported in other cultural and linguistic groups (Fasfous et al., 2013). These differences may reflect lower familiarity with standardized or timed testing, greater emphasis on accuracy over speed in non-western cultures, and variations in educational systems and approaches across cultural groups (Ardila, 1995; Ardila et al., 2002; Hayden et al., 2014). The proposed RBNs for Russian-speakers have identified fewer individuals as performing in the below average and exceptionally low range (T-score ≤36) compared to the CTT manual norms, which would be expected given no documented evidence of cognitive impairment in these participants.

Although CTT is construed a culture-fair test (D'Elia et al., 1996; Elkin-Frankston et al., 2007; Hartman-Maeir et al., 2008; Maj et al., 1993; Rabin et al., 2005), it is not fully equivalent across cultures (Agranovich et al., 2011; Agranovich & Puente, 2007; Fasfous et al., 2013; Melikyan et al., 2021), necessitating the development of culture-specific norms. Most of such norms are presented as means and standard deviations of raw scores stratified by age and education—the most salient contributors to CTT performance (Al-Joudi et al., 2019; Avila et al., 2019; Gaudreau et al., 2022; Hsieh & Tori, 2007; Indorewalla et al., 2022; Konstantopoulos et al., 2013; Kudiaki & Aslan, 2008; La Rue et al., 1999; Messinis et al., 2011; Pontón et al., 1996; Sant’ Ana Rabelo et al., 2010; Vogel et al., 2013). This study presents regression-based CTT norms, following examples set for TMT (Cavaco et al., 2013; Espenes et al., 2020; Suarez et al., 2021; Testa et al., 2009). Regression-based norms offer the advantage of more precisely aligning with an individual’s demographic characteristics while reducing instability in estimates derived from small sample subgroups. The present study contributes to a limited body of literature demonstrating that the use of culture-specific norms, as opposed to manual norms, can improve the accuracy of scores’ interpretation.

This normative study has several implications for clinical practice. Although it is recognized that the use of psychometric measures developed for North American English speakers may result in misclassification when applied to individuals from other cultural and linguistic backgrounds (Ardila et al., 2002; Staios et al., 2023), the lack of culturally appropriate tests and norms often leaves neuropsychologists with limited options when assessing culturally dissimilar individuals. The normative data reported in the present study may help improve diagnostic validity in the assessment of Russian-speakers and reduce the risk of misclassification.

It is important to note that present norms are intended for individuals of any ethnic or cultural background who grew up in the territory of the former Soviet Union (during Soviet or post-Soviet times) and for whom Russian is the native language. Despite the cultural diversity of populations across the post-Soviet space, these groups shared systems of governance, healthcare, education, employment, mass communication, culture, and an official language (Russian) for approximately seven decades.

For individuals who immigrated from Soviet Union or from post-Soviet space during childhood, and who subsequently completed their education and work experience in their host country, local norms (e.g., United States or other culture-specific norms) may be more appropriate due to their quasi complete immersion in the local language and culture (Dietrich & Hernandez, 2019). To that point, previous work showed that CTT raw scores decreased with increased acculturation among Russian-speaking immigrants living in the United States. (Gorenstein, 2022). Therefore, the degree of acculturation should be taken into account when choosing norms and interpreting the results, as acculturation might influence test performance. Furthermore, caution is warranted when assessing individuals who grew up in the post-Soviet space after the dissolution of the Soviet Union outside the Russian Federation, were primarily immersed in their country’s native culture and language, and for whom Russian is not the primary language. Previous studies indicated that applying normative data derived from one cultural and linguistic group to other groups—even those that appear culturally similar—may still lead to misclassification (Duggan et al., 2019; Staios et al., 2023).

### Strengths and limitations

This study has several strengths. First, our normative sample is fairly representative of the adult population in Russia. Participants were recruited from big cities and small towns among several regions of Russia, from wide range of educational backgrounds and age groups to ensure appropriate demographic representation. The sample’s mean age of 37 years is close to the reported 39 years average for the population of Russia, and the 54% women also closely matches the demographics of the Russian population. Of note, the study sample is more educated than average, with 52% having at least a university degree, compared to 23% with a university degree or higher in the Russian population (Federal agency of state statistics [Federalnaia sluzhba gosudarstvennoi statistiki], 2010). To ensure cultural appropriateness of the tests, testing materials were carefully translated in accordance with the established guidelines. Finally, a regression-based, rather than conventional, approach to the norm development was utilized to ensure a closer match to the demographic characteristics for each individual.

We acknowledge several limitations. First, the normative sample size is relatively small. However, the use of a regression-based approach for the development of norms mitigates this potential shortcoming, as it has been described as appropriate for smaller sample sizes (Stevens, 2002). Further studies with larger sample size would help confirm and expand upon the data. Second, intact cognitive abilities of original studies’ participants were determined using self-report and not a more objective screening test, which was not available in Russian at the time of data collection. Yet, the standardized approach to screening with a comprehensive structured health questionnaire is believed to be sufficient to confirm grossly intact cognition in recruited participants (Anzai et al., 2023).

### Conclusion

Russian-speaking individuals who grew up in the Soviet Union or post-Soviet space constitute a large population both within Russia and internationally, yet neuropsychological tests and normative data tailored to this group remain critically limited. The present study addresses this gap by providing regression-based Color Trails Test norms derived from a representative sample of neurologically healthy Russian-speaking adults, thereby strengthening diagnostic accuracy and advancing neuropsychological assessment for this population.

## Supplementary Material

Supplementary_materials_Revision_NoHighlight_acag035

## Data Availability

The dataset used for the current study is available to qualified researchers from the corresponding author on a reasonable request.
