# Supplementary material for: Color Trails Test: regression-based norms for Russian-speakers
Source: Arch Clin Neuropsychol. 2026 May 20;41(4):acag035. doi: 10.1093/arclin/acag035 (PMC13189164; doi:10.1093/arclin/acag035)
Supplement: Supplementary_materials_Revision_NoHighlight_acag035 [file supplementary_materials_revision_nohighlight_acag035.docx]

**Supplementary materials**


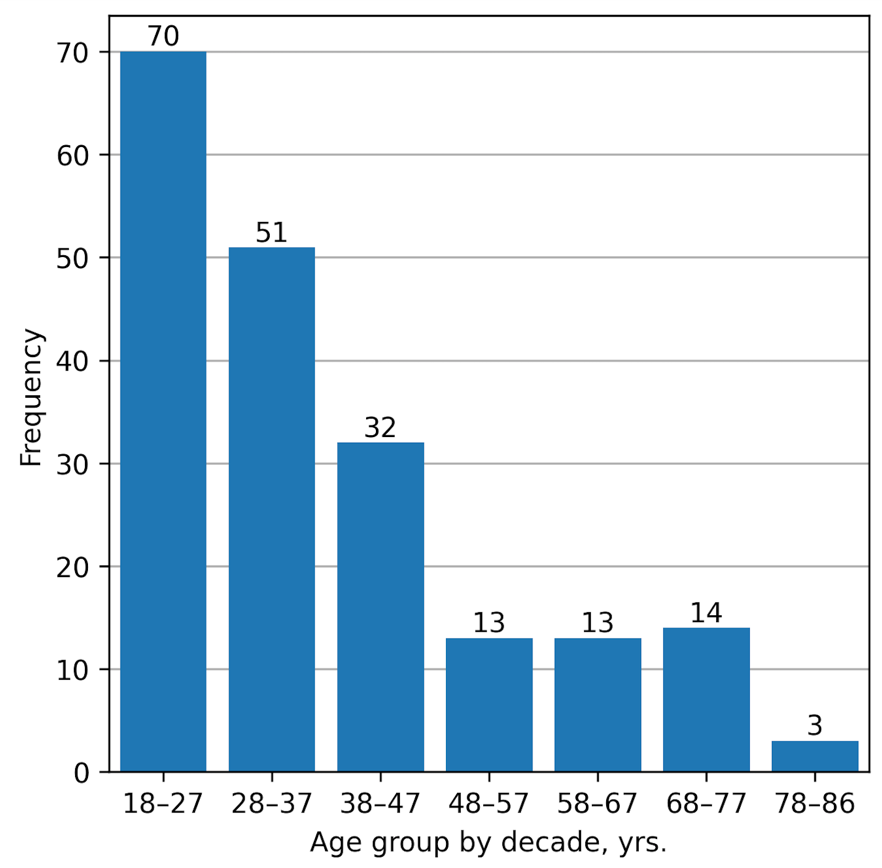


**Supplementary figure 1.** Distribution of participants’ age by decade

| **Supplementary table 1.** Characteristics of participants with settlement data available by settlement type | | |
| --- | --- | --- |
|  | **Urban** | **Rural** |
|  | **N (%)** | |
| N | 49 (25) | 52 (27) |
| Sex  Men  Women | 21 (43)  28 (57) | 21 (40)  31 (60) |
| Education  Below university  University or higher | 17 (35)  32 (65) | 30 (58)  22 (42) |
|  | **Mean (SD) [range]** | |
| Age, y | 36.5 (14.5) [19.0-73.0] | 49.2 (19.3) [18.0-86.0] |
| University education in Russia is equivalent to a master’s or professional (e.g., MD, JD) degree. | | |

| **Supplementary table 2.** Multiple regressions predicting CTT1 and CTT2 scaled scores by settlement | | | | |
| --- | --- | --- | --- | --- |
| **Coefficient** | **CTT1, N = 101** | | **CTT2, N = 100** | |
|  | **SS (95% CI)** | ***p*-value** | **SS (95% CI)** | ***p*-value** |
| Intercept | 12.768 (11.150, 14.386) | <0.001 | 12.588 (11.280, 13.895) | <0.001 |
| Education, less than university degree | Ref | - | Ref | - |
| Education, university degree or greater | 0.862 (-0.283, 2.008) | 0.139 | 0.567 (-0.360, 1.495) | 0.228 |
| Settlement, urban | Ref | - | Ref | - |
| Settlement, rural | -0.954 (-2.172, 0.264) | 0.123 | -1.604 (-2.585, -0.622) | 0.002 |
| Age, yrs. | -0.076 (-0.109, -0.043) | <0.001 | -0.070 (-0.097, -0.044) | <0.001 |
| SEE | 2.001 | - | 1.610 | - |
| R² | 0.278 | - | 0.389 | - |
| CTT=Color Trails Test; CI=Confidence Interval; SEE=Standard Error of the Estimate. One participant was excluded from the CTT2 analysis only due to missing data | | | | |
